# *N*,*N*′-Bis(4-amino­benz­yl)oxalamide

**DOI:** 10.1107/S1600536811000882

**Published:** 2011-01-15

**Authors:** Juan Saulo Gonzalez-Gonzalez, Francisco J. Martínez-Martínez, Efrén V. García-Báez, Olivia M. Franco-Hernández, Itzia I. Padilla-Martínez

**Affiliations:** a Facultad de Ciencias Químicas, Universidad de Colima, Carretera Coquimatlán-Colima, Coquimatlán Colima, Mexico 28400; b Unidad Profesional Interdisciplinaria de Biotecnología, Instituto Politécnico Nacional, Avenida Acueducto s/n, Barrio La Laguna Ticomán, México DF 07340, Mexico

## Abstract

In the title compound, C_16_H_18_N_4_O_2_, the two carbonyl groups are in an anti­periplanar conformation with an O=C—C=O torsion angle of 173.86 (17)°. In the crystal, a pair of inter­molecular N—H⋯O hydrogen bonds, forming an *R*_2_^2^(10) ring motif, connect the mol­ecules into an inversion dimer. The dimers are further linked by N—H⋯N and C—H⋯π inter­actions, forming a zigzag chain along the *b* axis.

## Related literature

For bond-length data, see: Allen *et al.* (1987 ▶). For hydrogen-bond motifs, see: Bernstein *et al.* (1995 ▶). For related structures, see: Lee & Wang (2007 ▶). For background to and applications of oxalamides, see: Martínez-Martínez *et al.* (1998 ▶); Padilla-Martínez *et al.* (2001 ▶); Nguyen *et al.* (2001 ▶).
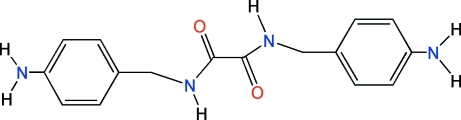


## Experimental

### 

#### Crystal data

C_16_H_18_N_4_O_2_
*M* *_r_* = 298.34
Monoclinic, 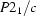

*a* = 10.7970 (9) Å
*b* = 8.0930 (8) Å
*c* = 17.9888 (7) Å
β = 110.151 (10)°
*V* = 1475.7 (2) Å^3^
*Z* = 4
Mo *K*α radiation
μ = 0.09 mm^−1^
*T* = 293 K
0.40 × 0.30 × 0.20 mm

#### Data collection

Bruker APEXII area-detector diffractometer
13460 measured reflections
2577 independent reflections
2041 reflections with *I* > 2σ(*I*)
*R*_int_ = 0.050

#### Refinement

*R*[*F* ^2^ > 2σ(*F* ^2^)] = 0.047
*wR*(*F* ^2^) = 0.141
*S* = 1.05
2577 reflections
199 parameters
H-atom parameters constrained
Δρ_max_ = 0.24 e Å^−3^
Δρ_min_ = −0.21 e Å^−3^

### 

Data collection: *APEX2* (Bruker, 2004 ▶); cell refinement: *SAINT* (Bruker, 2004 ▶); data reduction: *SAINT*; program(s) used to solve structure: *SHELXS97* (Sheldrick, 2008 ▶); program(s) used to refine structure: *SHELXL97* (Sheldrick, 2008 ▶); molecular graphics: *SHELXTL* (Sheldrick, 2008 ▶); software used to prepare material for publication: *SHELXL97* and *WinGX* (Farrugia, 1999 ▶).

## Supplementary Material

Crystal structure: contains datablocks I, global. DOI: 10.1107/S1600536811000882/is2647sup1.cif

Structure factors: contains datablocks I. DOI: 10.1107/S1600536811000882/is2647Isup2.hkl

Additional supplementary materials:  crystallographic information; 3D view; checkCIF report

## Figures and Tables

**Table 1:** Hydrogen-bond geometry (Å, °) *Cg*1 is the centroid of the C1–C6 ring.

*D*—H⋯*A*	*D*—H	H⋯*A*	*D*⋯*A*	*D*—H⋯*A*
N4—H4*A*⋯N16^i^	0.86	2.48	3.240 (3)	147
N4—H4*B*⋯O9^ii^	0.86	2.35	3.196 (2)	170
N8—H8⋯N4^iii^	0.86	2.31	3.085 (2)	150
N11—H11⋯O9^iv^	0.86	2.27	3.015 (2)	145
C6—H6⋯*Cg*1^iii^	0.93	2.94	3.836 (3)	162

Symmetry codes: (i) 
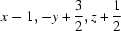
; (ii) 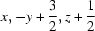; (iii) 
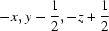
; (iv) 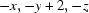.

**Fig. 1.**
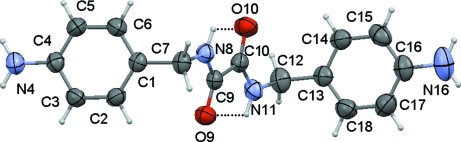
Molecular structure of the title compound, showing the atom labeling scheme and 50% probability displacement ellipsoids. Dashed lines indicate intramolecular hydrogen bonding.

**Fig. 2.**
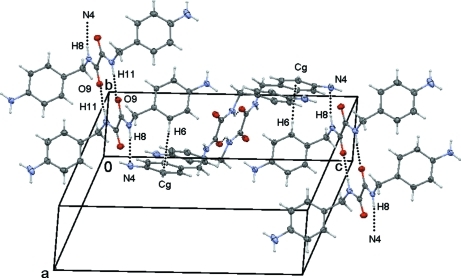
Packing diagram of the title compound, dashed lines indicate intermolecular hydrogen bonding.

## supplementary crystallographic information

### Comment

The chemical structure of oxalamides favors the formation of intra and intermolecular hydrogen bonding interactions (Martínez-Martínez *et al.*, 1998; Padilla-Martínez *et al.*, 2001; Nguyen *et al.*, 2001). Herein we present the title compound, (I), a new bis-oxalamide.

The title compound (I) forms monoclinic crystals (*P*2_1_/*c*, *Z* = 4). Carbonyl groups are antiperiplanar, with an O9—C9—C10—O10 torsion angle of 173.86 (17)°. The oxalamide group is almost planar, with an N8—C9—C10—N11 torsion angle of 171.92 (17)°. The aminobenzyl groups are twisted by 69.43 (5)° (C1—C7/N4) and 73.78 (5)° (C13—C18/N16) out of the oxalamide group mean plane (C7/N8/C9/O9/C10/O10/N11/C12) and are almost parallel to each other with an angle between the planes of 4.56 (5)°. According to graph-set notation (Bernstein *et al.*, 1995), two S(5) rings are formed through N8—H8···O10 and N11—H11···O9 interactions. N···O distances and N—H···O angles are in the range for strong hydrogen bonding, in agreement with similar structures (Lee and Wang, 2007). The zero dimensional array is given by pairing of two molecules through self complementary strong N11—H11···O9 hydrogen bonding, to form the *R*^2^_2_(10) motif characteristic of oxalamides. The molecules are connected by N8—H8···N4 and C6—H6···*Cg*1 into a zigzag chain running along the *b* axis; *Cg*1 is the centroid of the C1–C6 ring. Amine N4—H4A···N16 and N4—H4B···O9 hydrogen bonding interactions give the second and third dimensions, forming C(17) and C(10) chains, respectively.

### Experimental

A mixture of diethyl oxalate (2 ml,14 mmol) and 4-aminobenzylamine (3.34 ml, 28 mmol) in ethanol (30 ml) was refluxed for 6 h. The suspension was filtered and the resulting solid was washed with cold ethyl alcohol to yield 3.25 g (73%) of a pale yellow solid (m.p. 250–252 °C).

IR (neat, cm^-1^): 3415, 3346, 3202, 3038, 1651. Anal. calcd. for C_16_H_18_N_4_O_2_: C 64.41, H 6.08, N 18.78%; found: C 64.07, H 6.25, N 18.44%. ^1^H NMR (300 MHz, DMSO_d-6_, δ) CH_2_ 4.11 (d, 2H), NH 9.03 (t, 1H), Aromatics: 6.46 (d, 2H), 6.90 (d, 2H), NH_2_ 4.95 (s, 2H). ^13^C NMR (75.46 MHz, DMSO_d-6_, δ) 42.7, 114.2, 126.3, 129.1, 148.3, 160.5. ESI MS = calc. m/*z* 298.14, found m/*z* 320.8 [*M* + Na]^+^.

### Refinement

H atoms bonded to C were positioned geometrically with aromatic C—H = 0.93 Å and aliphatic C—H = 0.97 Å. Their displacement parameters were set at *U*_iso_(H)= 1.2*U*_eq_(C). The amino H atoms were found in a Fourier difference map and refined with the constraints of N—H = 0.86 Å and *U*_iso_(H) = 1.2*U*_eq_(N).

### Crystal data

**Table:**

C_16_H_18_N_4_O_2_	*F*(000) = 632
*M**_r_* = 298.34	*D*_x_ = 1.343 Mg m^−^^3^
Monoclinic, *P*2_1_/*c*	Mo *K*α radiation, λ = 0.71073 Å
Hall symbol: -P 2ybc	Cell parameters from 600 reflections
*a* = 10.7970 (9) Å	θ = 20–25°
*b* = 8.0930 (8) Å	µ = 0.09 mm^−^^1^
*c* = 17.9888 (7) Å	*T* = 293 K
β = 110.151 (10)°	Prism, colorless
*V* = 1475.7 (2) Å^3^	0.40 × 0.30 × 0.20 mm
*Z* = 4	

### Data collection

**Table:**

Bruker APEXII area-detector diffractometer	2041 reflections with *I* > 2σ(*I*)
Radiation source: fine-focus sealed tube	*R*_int_ = 0.050
graphite	θ_max_ = 25.0°, θ_min_ = 2.4°
φ and ω scans	*h* = −12→12
13460 measured reflections	*k* = −8→9
2577 independent reflections	*l* = −21→21

### Refinement

**Table:**

Refinement on *F*^2^	Primary atom site location: structure-invariant direct methods
Least-squares matrix: full	Secondary atom site location: difference Fourier map
*R*[*F*^2^ > 2σ(*F*^2^)] = 0.047	Hydrogen site location: inferred from neighbouring sites
*wR*(*F*^2^) = 0.141	H-atom parameters constrained
*S* = 1.05	*w* = 1/[σ^2^(*F*_o_^2^) + (0.0744*P*)^2^ + 0.3652*P*] where *P* = (*F*_o_^2^ + 2*F*_c_^2^)/3
2577 reflections	(Δ/σ)_max_ < 0.001
199 parameters	Δρ_max_ = 0.24 e Å^−^^3^
0 restraints	Δρ_min_ = −0.21 e Å^−^^3^

### Special details

**Table:**

Geometry. Bond distances, angles *etc*. have been calculated using the rounded fractional coordinates. All su's are estimated from the variances of the (full) variance-covariance matrix. The cell e.s.d.'s are taken into account in the estimation of distances, angles and torsion angles
Refinement. Refinement of *F*^2^ against ALL reflections. The weighted *R*-factor *wR* and goodness of fit *S* are based on *F*^2^, conventional *R*-factors *R* are based on *F*, with *F* set to zero for negative *F*^2^. The threshold expression of *F*^2^ > σ(*F*^2^) is used only for calculating *R*-factors(gt) *etc*. and is not relevant to the choice of reflections for refinement. *R*-factors based on *F*^2^ are statistically about twice as large as those based on *F*, and *R*- factors based on ALL data will be even larger.

### Fractional atomic coordinates and isotropic or equivalent isotropic displacement parameters (Å^2^)

**Table:**

	*x*	*y*	*z*	*U*_iso_*/*U*_eq_	
O9	−0.04287 (12)	0.80255 (17)	0.03285 (8)	0.0510 (4)	
O10	0.28644 (13)	0.6703 (2)	0.11061 (9)	0.0636 (5)	
N4	−0.22384 (17)	0.7608 (2)	0.35278 (10)	0.0581 (6)	
N8	0.04232 (14)	0.56568 (19)	0.09827 (9)	0.0439 (5)	
N11	0.19920 (15)	0.8886 (2)	0.03145 (9)	0.0478 (5)	
N16	0.4741 (2)	0.6082 (3)	−0.21045 (14)	0.0872 (9)	
C1	−0.12077 (16)	0.5601 (2)	0.16642 (10)	0.0404 (6)	
C2	−0.22418 (18)	0.6700 (3)	0.15269 (11)	0.0488 (6)	
C3	−0.26137 (18)	0.7320 (3)	0.21331 (11)	0.0505 (7)	
C4	−0.19297 (17)	0.6871 (2)	0.29106 (11)	0.0431 (6)	
C5	−0.09016 (19)	0.5751 (3)	0.30558 (11)	0.0493 (6)	
C6	−0.05570 (18)	0.5125 (3)	0.24415 (11)	0.0488 (6)	
C7	−0.08159 (18)	0.4955 (3)	0.09935 (11)	0.0472 (6)	
C9	0.05074 (17)	0.7099 (2)	0.06609 (10)	0.0399 (6)	
C10	0.19201 (18)	0.7547 (2)	0.07215 (10)	0.0430 (6)	
C12	0.3227 (2)	0.9461 (3)	0.02409 (13)	0.0561 (7)	
C13	0.36006 (17)	0.8540 (2)	−0.03788 (11)	0.0453 (6)	
C14	0.45884 (18)	0.7383 (3)	−0.01763 (13)	0.0542 (7)	
C15	0.4959 (2)	0.6559 (3)	−0.07410 (15)	0.0610 (8)	
C16	0.4330 (2)	0.6865 (3)	−0.15412 (13)	0.0563 (7)	
C17	0.3328 (2)	0.8023 (3)	−0.17473 (13)	0.0667 (8)	
C18	0.2968 (2)	0.8838 (3)	−0.11778 (13)	0.0600 (7)	
H2	−0.27013	0.70323	0.10101	0.0586*	
H3	−0.33254	0.80416	0.20198	0.0606*	
H4A	−0.28523	0.83393	0.34251	0.0696*	
H4B	−0.18144	0.73296	0.40102	0.0696*	
H5	−0.04405	0.54185	0.35724	0.0591*	
H6	0.01287	0.43645	0.25513	0.0586*	
H7A	−0.15096	0.52059	0.04962	0.0567*	
H7B	−0.07300	0.37623	0.10376	0.0567*	
H8	0.11364	0.51010	0.11973	0.0527*	
H11	0.12828	0.94390	0.00844	0.0574*	
H12A	0.31459	1.06273	0.01087	0.0673*	
H12B	0.39291	0.93392	0.07483	0.0673*	
H14A	0.50224	0.71438	0.03561	0.0650*	
H15	0.56389	0.57895	−0.05811	0.0732*	
H16A	0.43692	0.63146	−0.25982	0.1046*	
H16B	0.53677	0.53665	−0.19591	0.1046*	
H17	0.28877	0.82581	−0.22794	0.0801*	
H18	0.22843	0.96034	−0.13353	0.0720*	

### Atomic displacement parameters (Å^2^)

**Table:**

	*U*^11^	*U*^22^	*U*^33^	*U*^12^	*U*^13^	*U*^23^
O9	0.0456 (7)	0.0494 (8)	0.0576 (8)	0.0014 (6)	0.0173 (6)	0.0056 (6)
O10	0.0457 (8)	0.0741 (10)	0.0721 (10)	0.0067 (7)	0.0219 (7)	0.0212 (8)
N4	0.0659 (11)	0.0607 (11)	0.0553 (10)	0.0091 (9)	0.0308 (9)	−0.0015 (8)
N8	0.0436 (8)	0.0456 (9)	0.0468 (9)	0.0005 (7)	0.0212 (7)	0.0029 (7)
N11	0.0457 (9)	0.0467 (9)	0.0575 (9)	−0.0019 (7)	0.0261 (7)	0.0031 (8)
N16	0.0781 (13)	0.1039 (18)	0.1004 (16)	−0.0224 (13)	0.0575 (12)	−0.0310 (14)
C1	0.0394 (9)	0.0395 (10)	0.0437 (10)	−0.0073 (7)	0.0163 (8)	−0.0001 (8)
C2	0.0422 (10)	0.0619 (13)	0.0405 (10)	0.0012 (9)	0.0119 (8)	0.0086 (9)
C3	0.0406 (10)	0.0573 (12)	0.0553 (12)	0.0093 (9)	0.0187 (9)	0.0087 (10)
C4	0.0428 (10)	0.0425 (10)	0.0489 (10)	−0.0055 (8)	0.0220 (8)	0.0014 (8)
C5	0.0525 (11)	0.0547 (12)	0.0403 (10)	0.0066 (9)	0.0156 (8)	0.0079 (9)
C6	0.0480 (10)	0.0468 (11)	0.0526 (11)	0.0088 (9)	0.0186 (9)	0.0036 (9)
C7	0.0497 (10)	0.0462 (11)	0.0500 (11)	−0.0096 (9)	0.0226 (9)	−0.0050 (9)
C9	0.0432 (10)	0.0431 (10)	0.0362 (9)	−0.0010 (8)	0.0173 (8)	−0.0044 (8)
C10	0.0442 (10)	0.0477 (11)	0.0399 (9)	−0.0013 (9)	0.0179 (8)	−0.0017 (8)
C12	0.0536 (12)	0.0529 (12)	0.0691 (13)	−0.0151 (9)	0.0305 (10)	−0.0055 (10)
C13	0.0384 (9)	0.0474 (11)	0.0541 (11)	−0.0074 (8)	0.0210 (8)	0.0058 (9)
C14	0.0426 (10)	0.0638 (13)	0.0574 (12)	−0.0010 (10)	0.0187 (9)	0.0143 (10)
C15	0.0513 (12)	0.0529 (13)	0.0876 (16)	0.0037 (10)	0.0351 (11)	0.0096 (12)
C16	0.0477 (11)	0.0610 (13)	0.0701 (14)	−0.0178 (10)	0.0329 (10)	−0.0091 (11)
C17	0.0548 (12)	0.0921 (18)	0.0524 (12)	−0.0055 (12)	0.0174 (10)	0.0059 (12)
C18	0.0485 (11)	0.0689 (14)	0.0639 (13)	0.0098 (10)	0.0210 (10)	0.0134 (11)

### Geometric parameters (Å, °)

**Table:**

O9—C9	1.235 (2)	C9—C10	1.535 (3)
O10—C10	1.224 (2)	C12—C13	1.507 (3)
N4—C4	1.398 (3)	C13—C18	1.384 (3)
N8—C7	1.460 (3)	C13—C14	1.371 (3)
N8—C9	1.320 (2)	C14—C15	1.385 (3)
N11—C10	1.325 (2)	C15—C16	1.386 (3)
N11—C12	1.461 (3)	C16—C17	1.382 (3)
N16—C16	1.391 (3)	C17—C18	1.382 (3)
N4—H4A	0.8600	C2—H2	0.9300
N4—H4B	0.8600	C3—H3	0.9300
N8—H8	0.8600	C5—H5	0.9300
N11—H11	0.8600	C6—H6	0.9300
N16—H16A	0.8600	C7—H7A	0.9700
N16—H16B	0.8600	C7—H7B	0.9700
C1—C6	1.386 (3)	C12—H12A	0.9700
C1—C7	1.503 (3)	C12—H12B	0.9700
C1—C2	1.382 (3)	C14—H14A	0.9300
C2—C3	1.380 (3)	C15—H15	0.9300
C3—C4	1.386 (3)	C17—H17	0.9300
C4—C5	1.386 (3)	C18—H18	0.9300
C5—C6	1.378 (3)		
			
O9···N11	2.713 (2)	H2···C14^xi^	2.9800
O9···N11^i^	3.015 (2)	H2···H7A	2.3500
O9···N4^ii^	3.196 (2)	H2···H14A^xi^	2.3300
O10···C13	3.382 (2)	H3···H4A	2.4100
O10···N8	2.704 (2)	H3···C18^i^	3.0400
O9···H11^i^	2.2700	H4A···H3	2.4100
O9···H5^iii^	2.6900	H4A···O10^iii^	2.8500
O9···H4B^ii^	2.3500	H4A···H8^iii^	2.2500
O9···H7A	2.6300	H4A···N16^vii^	2.4800
O9···H11	2.3400	H4A···C16^vii^	3.0700
O10···H8	2.3200	H4A···H16B^vii^	2.0900
O10···H12B	2.6100	H4B···H5	2.4500
O10···H4A^iv^	2.8500	H4B···H8^iii^	2.4300
O10···H16B^v^	2.6000	H4B···O9^vi^	2.3500
O10···H16A^vi^	2.8300	H5···H4B	2.4500
O10···H17^vi^	2.9000	H5···O9^iv^	2.6900
N4···N8^iii^	3.085 (2)	H5···C9^iv^	3.0300
N4···O9^vi^	3.196 (2)	H6···C3^iv^	3.0300
N4···N16^vii^	3.240 (3)	H7A···O9	2.6300
N8···O10	2.704 (2)	H7A···H2	2.3500
N8···N4^iv^	3.085 (2)	H7A···C10^ix^	3.0500
N11···O9^i^	3.015 (2)	H8···O10	2.3200
N11···O9	2.713 (2)	H8···N4^iv^	2.3100
N16···N4^viii^	3.240 (3)	H8···C4^iv^	3.0300
N4···H16B^vii^	2.9300	H8···H4A^iv^	2.2500
N4···H8^iii^	2.3100	H8···H4B^iv^	2.4300
N16···H4A^viii^	2.4800	H11···O9	2.3400
C3···C18^i^	3.509 (3)	H11···O9^i^	2.2700
C7···C10^ix^	3.536 (3)	H12A···H18	2.5800
C7···C9^ix^	3.523 (3)	H12A···C14^x^	2.9000
C9···C7^ix^	3.523 (3)	H12A···C15^x^	3.0100
C10···C7^ix^	3.536 (3)	H12B···O10	2.6100
C12···C14^x^	3.506 (3)	H12B···H14A	2.3700
C13···O10	3.382 (2)	H14A···C2^xii^	3.0000
C14···C15^v^	3.547 (3)	H14A···H2^xii^	2.3300
C14···C12^x^	3.506 (3)	H14A···H12B	2.3700
C15···C14^v^	3.547 (3)	H14A···C15^v^	3.0700
C18···C3^i^	3.509 (3)	H14A···H15^v^	2.5500
C2···H18^i^	3.0100	H15···H16B	2.4200
C2···H14A^xi^	3.0000	H15···C14^v^	2.9600
C3···H18^i^	2.9600	H15···H14A^v^	2.5500
C3···H6^iii^	3.0300	H16A···H17	2.4500
C4···H8^iii^	3.0300	H16A···O10^ii^	2.8300
C9···H5^iii^	3.0300	H16B···H15	2.4200
C10···H7A^ix^	3.0500	H16B···O10^v^	2.6000
C14···H2^xii^	2.9800	H16B···N4^viii^	2.9300
C14···H15^v^	2.9600	H16B···H4A^viii^	2.0900
C14···H12A^x^	2.9000	H17···H16A	2.4500
C15···H14A^v^	3.0700	H17···O10^ii^	2.9000
C15···H12A^x^	3.0100	H18···H12A	2.5800
C16···H4A^viii^	3.0700	H18···C2^i^	3.0100
C18···H3^i^	3.0400	H18···C3^i^	2.9600
			
C7—N8—C9	123.50 (17)	C13—C14—C15	121.9 (2)
C10—N11—C12	122.67 (17)	C14—C15—C16	120.9 (2)
H4A—N4—H4B	120.00	N16—C16—C17	122.0 (2)
C4—N4—H4B	120.00	N16—C16—C15	120.7 (2)
C4—N4—H4A	120.00	C15—C16—C17	117.3 (2)
C7—N8—H8	118.00	C16—C17—C18	121.3 (2)
C9—N8—H8	118.00	C13—C18—C17	121.5 (2)
C10—N11—H11	119.00	C1—C2—H2	119.00
C12—N11—H11	119.00	C3—C2—H2	119.00
C16—N16—H16A	120.00	C2—C3—H3	120.00
C16—N16—H16B	120.00	C4—C3—H3	120.00
H16A—N16—H16B	120.00	C4—C5—H5	120.00
C2—C1—C6	117.19 (17)	C6—C5—H5	120.00
C6—C1—C7	121.88 (17)	C1—C6—H6	119.00
C2—C1—C7	120.93 (16)	C5—C6—H6	119.00
C1—C2—C3	122.01 (18)	N8—C7—H7A	109.00
C2—C3—C4	120.2 (2)	N8—C7—H7B	109.00
N4—C4—C5	121.58 (17)	C1—C7—H7A	109.00
C3—C4—C5	118.37 (18)	C1—C7—H7B	109.00
N4—C4—C3	119.97 (17)	H7A—C7—H7B	108.00
C4—C5—C6	120.60 (18)	N11—C12—H12A	109.00
C1—C6—C5	121.6 (2)	N11—C12—H12B	109.00
N8—C7—C1	112.83 (17)	C13—C12—H12A	109.00
O9—C9—N8	125.51 (18)	C13—C12—H12B	109.00
O9—C9—C10	121.24 (15)	H12A—C12—H12B	108.00
N8—C9—C10	113.25 (15)	C13—C14—H14A	119.00
O10—C10—C9	121.48 (16)	C15—C14—H14A	119.00
N11—C10—C9	113.58 (16)	C14—C15—H15	120.00
O10—C10—N11	124.94 (19)	C16—C15—H15	120.00
N11—C12—C13	113.26 (18)	C16—C17—H17	119.00
C12—C13—C18	121.34 (18)	C18—C17—H17	119.00
C12—C13—C14	121.55 (18)	C13—C18—H18	119.00
C14—C13—C18	117.10 (19)	C17—C18—H18	119.00
			
C7—N8—C9—C10	−179.47 (15)	C4—C5—C6—C1	0.8 (3)
C9—N8—C7—C1	−83.4 (2)	O9—C9—C10—O10	173.86 (17)
C7—N8—C9—O9	−0.5 (3)	O9—C9—C10—N11	−7.1 (2)
C12—N11—C10—O10	3.8 (3)	N8—C9—C10—O10	−7.1 (2)
C10—N11—C12—C13	80.5 (2)	N8—C9—C10—N11	171.93 (15)
C12—N11—C10—C9	−175.23 (16)	N11—C12—C13—C14	−105.0 (2)
C2—C1—C6—C5	−1.6 (3)	N11—C12—C13—C18	75.6 (2)
C7—C1—C2—C3	−179.5 (2)	C12—C13—C14—C15	−178.5 (2)
C6—C1—C7—N8	−72.8 (2)	C18—C13—C14—C15	1.0 (3)
C7—C1—C6—C5	178.5 (2)	C12—C13—C18—C17	178.5 (2)
C6—C1—C2—C3	0.6 (3)	C14—C13—C18—C17	−0.9 (3)
C2—C1—C7—N8	107.3 (2)	C13—C14—C15—C16	−0.6 (4)
C1—C2—C3—C4	1.3 (3)	C14—C15—C16—N16	177.4 (2)
C2—C3—C4—N4	174.6 (2)	C14—C15—C16—C17	0.2 (4)
C2—C3—C4—C5	−2.1 (3)	N16—C16—C17—C18	−177.4 (2)
N4—C4—C5—C6	−175.6 (2)	C15—C16—C17—C18	−0.1 (4)
C3—C4—C5—C6	1.1 (3)	C16—C17—C18—C13	0.5 (4)

Symmetry codes: (i) −*x*, −*y*+2, −*z*; (ii) *x*, −*y*+3/2, *z*−1/2; (iii) −*x*, *y*+1/2, −*z*+1/2; (iv) −*x*, *y*−1/2, −*z*+1/2; (v) −*x*+1, −*y*+1, −*z*; (vi) *x*, −*y*+3/2, *z*+1/2; (vii) *x*−1, −*y*+3/2, *z*+1/2; (viii) *x*+1, −*y*+3/2, *z*−1/2; (ix) −*x*, −*y*+1, −*z*; (x) −*x*+1, −*y*+2, −*z*; (xi) *x*−1, *y*, *z*; (xii) *x*+1, *y*, *z*.

### Hydrogen-bond geometry (Å, °)

**Table:**

*Cg*1 is the centroid of the C1–C6 ring.

**Table:**

*D*—H···*A*	*D*—H	H···*A*	*D*···*A*	*D*—H···*A*
N4—H4A···N16^vii^	0.86	2.48	3.240 (3)	147
N4—H4B···O9^vi^	0.86	2.35	3.196 (2)	170
N8—H8···O10	0.86	2.32	2.704 (2)	107
N8—H8···N4^iv^	0.86	2.31	3.085 (2)	150
N11—H11···O9	0.86	2.34	2.713 (2)	107
N11—H11···O9^i^	0.86	2.27	3.015 (2)	145
C6—H6···Cg1^iv^	0.93	2.94	3.836 (3)	162

Symmetry codes: (vii) *x*−1, −*y*+3/2, *z*+1/2; (vi) *x*, −*y*+3/2, *z*+1/2; (iv) −*x*, *y*−1/2, −*z*+1/2; (i) −*x*, −*y*+2, −*z*.
